# A model to predict the prognosis of diffuse large B-cell lymphoma based on ultrasound images

**DOI:** 10.1038/s41598-023-30533-y

**Published:** 2023-02-27

**Authors:** Wenjuan Lu, Wenqin Chen, Yasu Zhou, Ya Yuan, Hua Shu, Hongyan Deng, Xinhua Ye

**Affiliations:** Department of Ultrasound, The First Affiliated Hospital of Nanjing Medical University, 300 Guangzhou Road, Nanjing, 210029 Jiangsu China

**Keywords:** Cancer, Diseases, Oncology, Risk factors

## Abstract

The purpose of this paper was to assess the value of ultrasonography in the prognosis of diffuse large b-cell lymphoma (DLBCL) by developing a new prognostic model. One hundred and eleven DLBCL patients with complete clinical information and ultrasound findings were enrolled in our study. Univariate and multivariate regression analyses were used to identify independent risk factors for progression-free survival (PFS) and overall survival (OS). Receiver operator characteristic (ROC) curves were plotted and the corresponding area under the curve (AUC) was calculated to assess the accuracy of the international prognostic index (IPI) and new model in DLBCL risk stratification. The results suggested that hilum loss and ineffective treatment were independent risk variables for both PFS and OS in DLBCL patients. Additionally, the new model that added hilum loss and ineffective treatment to IPI had a better AUC for PFS and OS than IPI alone (AUC: 0.90, 0.88, and 0.82 vs. 0.71, 0.74, and 0.68 for 1-, 3-, and 5-year PFS, respectively; AUC: 0.92, 0.85 and 0.86 vs. 0.71, 0.75 and 0.76, for 1-, 3-, and 5-year OS, respectively). The model based on ultrasound images could better suggest PFS and OS of DLBCL, allowing for better risk stratification.

## Introduction

Diffuse large B-cell lymphoma (DLBCL) is one of the most common types of aggressive lymphomas, and it is highly heterogeneous both clinically and prognostically^1^. The 5-year survival rate for diffuse large B-cell lymphoma is about 60–70%^2^. 60% of DLBCL patients could be cured with 6–8 courses of R-CHOP chemotherapy, while 40% of patients continue to show chemo-refractory or relapse, and succumb to their disease, with a 2-year overall survival (OS) of 20% to 40%^2–4^. Generally, OS and progression-free survival (PFS) are common clinical indices used to indicate cancer survival in tumor patients. Statistically, OS has been considered as a critical indicator for monitoring and evaluating the effectiveness of cancer care. Progression-free survival (PFS), as surrogate endpoints, can be measured earlier and easier to assess than "true" endpoints^5^.

The International Prognostic Index (IPI), developed at the time of immunochemotherapy, identified four distinct risk groups and was the accepted prognostic tool^6^. In recent times, its ability to distinguish the first four risk groups has diminished due to the advent of rituximab^7^. Additionally, the revised IPI score (R-IPI) and expanded National Comprehensive Cancer Network (NCCN)-IPI have been developed to improve the prognostic power of the IPI^8^. In spite of the fact that both these scoring systems provided better prognostic guidance, they failed to identify subgroups of patients at very high risk^9^.

As an imaging examination, PET/CT was recommended for staging FDG-sensitive lymphomas^10^. Several studies have demonstrated that PET/CT was useful in predicting the PFS and OS of Non-Hodgkin's lymphoma at the end of chemotherapy^11,12^. What’s more, many other studies investigated new factors that affecting the prognosis and survival of DLBCL patients from various aspects such as morphology, Cell-of-origin classification, MYC rearrangement, and serological findings, thus establishing some risk models which presented better prediction of patient prognosis than IPI^13–15^. However, these studies ignored the fact that individual responses to chemotherapy vary greatly, and failed to visualize structural changes of the lesion dynamically in real time^16^. Hence, new or adjusted image prognostic tools are needed to provide more prognostic information.

Currently, ultrasound (US) imaging has good application prospects in the evaluation of the efficacy and prognosis of breast and gastrointestinal tumors. In lymphoma, US showed promise in the differential diagnosis of lymphoma from other lymph node diseases by assessing the long and short diameters, morphology, borders, internal echogenic features, hilum of lymph nodes as well as internal blood flow distribution. However, the conventional ultrasound was limited to the diagnosis of superficial lymphomas and few studies explored the value of US on the prognosis of DLBCL patients. The purpose of this study was to explore the value of US in the assessment of survival in patients with DLBCL by developing a new prognostic model. To our knowledge, no studies have linked US features to the prognosis of patients with DLBCL.

## Results

### Survival in DLBCL patients

**Figure 1:**
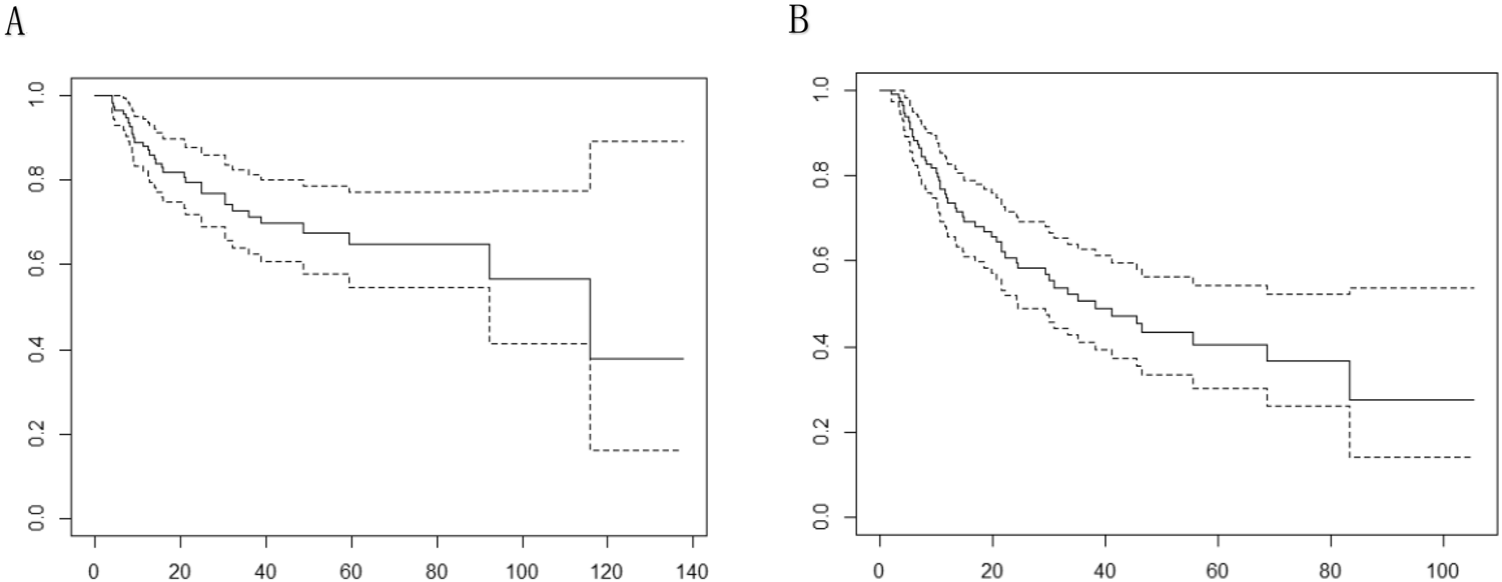
Kaplan–Meier curves for overall survival (**A**) and progression-free survival (**B**) of all enrolled patients.

Baseline characteristics of 111 DLBCL patients were showed in Table 1. In the 111 lymphoma patients receiving first-line standardized chemotherapy, 79% had an overall response, with 62% experiencing a complete response. 1-year PFS and OS rates were 74 ± 4.3% and 88 ± 3.1%, respectively, and the 3-year PFS and OS rates were respectively 51 ± 5.5% and 71 ± 4.8%. Similarly, the 5-year PFS and OS rates were 40 ± 6.1% and 65 ± 5.7%, respectively (Fig. 1).

**Table 1:** Baseline characteristics of 111 untreated DLBCL patients.

Features	Overall
	(N = 111)
Gender	
Female	61 (55.0%)
Male	50 (45.0%)
Age	
< 60	58 (52.3%)
> 60	53 (47.7%)
ECOG PS	
0–1	87 (78.4%)
2–4	24 (21.6%)
A/B	
A	71 (64.0%)
B	40 (36.0%)
Response assessment	
CR or PR	88 (79.3%)
SD or PD	23 (20.7%)
Stage	
I–II	28 (25.0%)
III–IV	83 (75.0%)
LT ratio	
> 2	33 (29.7%)
< 2	78 (70.3%)
Hilum loss	
No	29 (26.1%)
Yes	82 (73.9%)
Border	
Clear	95 (85.6%)
Unclear	16 (14.4%)
Blend	
No	48 (43.2%)
Yes	63 (56.8%)
IPI	
Score 0–2	56 (50.5%)
Score 3–5	55 (49.5%)
Extra-nodal involvement involvements	
0–1	39 (35.1%)
> 2	72 (64.9%)
β2-MG	
< 2.53	55 (49.5%)
> 2.53	56 (50.5%)
HB	
< 120 g/l	59 (53.2%)
> 120 g/l	52 (46.8%)
LDH	
< ULN^a^	46 (41.4%)
> ULN	65 (58.6%)

The tests used in this table were all chi-square test, Yates’ continuity correction test or Fisher’s exact test.

ECOG PS, Eastern Cooperative Oncology Group performance status; LDH, lactate dehydrogenase; β2-MG, β2-microglobulin; HB, hemoglobin; CR, complete remission; PD, progressive disease; PR, partial remission; SD, stable disease; ULN, upper limit of normal; L/T ratio: longitudinal to transverse (LT) nodal ratio.

^a^Normal value range ≤ 271 µ/L.

### Univariate analysis and multivariate regression analysis

Detailed univariate and multivariate Cox proportional hazards regression analysis of PFS and OS were shown in Tables 2 and 3. In the univariate analysis, ineffective treatment (SD or PD), advanced stage (III–IV), hilum loss, elevated LDH (> 271U/L), and decreased HB (< 120 g/L) were significantly associated with inferior outcomes in both PFS and OS. B symptom and unclear borders had a significant impact on PFS, but not OS. Extra-nodal involvements only affect the patient's OS.

**Table 2:** Analysis of clinicopathological features and survival in univariate models.

Features	Univariate analyses (PFS)		Univariate analyses (OS)	
	HR (95% CI)	*P* value	HR (95% CI)	*P* value
Male	1.172 (0.676–2.032)	0.561	0.743 (0.370–1.491)	0.409
Age > 60	1.092 (0.634–1.880)	0.748	1.087 (0.5383–2.195)	0.814
ECOG2-4	1.387 (0.698–2.755)	0.299	1.496 (0.610–3.671)	0.316
B symptom	1.893 (1.045–3.429)	0.018	1.924 (0.912–4.058)	0.055
SD or PD	7.617 (2.993–19.38)	< 0.001	5.710 (2.158–15.11)	< 0.001
Stage (III–IV)	4.203 (2.390–7.394)	< 0.001	2.927 (1.369–6.257)	0.033
LT ratio < 2	1.636 (0.909–2.944)	0.138	2.742 (1.269–5.924)	0.046
Hilum loss	2.309 (1.297–4.110)	0.017	4.147 (1.948–8.832)	0.010
Uncleared borders	3.566 (1.499–8.485)	< 0.001	1.984 (0.7391–5.324)	0.084
Blend	1.653 (0.965–2.832)	0.117	1.874 (0.935–3.756)	0.091
Extra-nodal involvement > 2	1.810 (0.948–3.455)	0.072	3.708(1.292–10.642)	0.015
β2-MG > 2.53	1.506 (0.869–2.611)	0.144	1.671 (0.8344–3.347)	0.144
HB < 120 g/l	2.181 (1.246–3.816)	0.006	2.334 (1.163–4.684)	0.018
LDH > ULN	3.005 (1.753–5.151)	< 0.001	3.502 (1.751–7.003)	0.002

CI, confidence interval; ECOG PS, Eastern Cooperative Oncology Group performance status; LDH, lactate dehydrogenase; β2-MG, β2-microglobulin; HB, hemoglobin; CR, complete remission; PD, progressive disease; PR, partial remission; SD, stable disease; ULN, upper limit of normal; L/T ratio: longitudinal to transverse (LT) nodal ratio; PFS, progression-free survival; OS, overall survival.

**Table 3:**
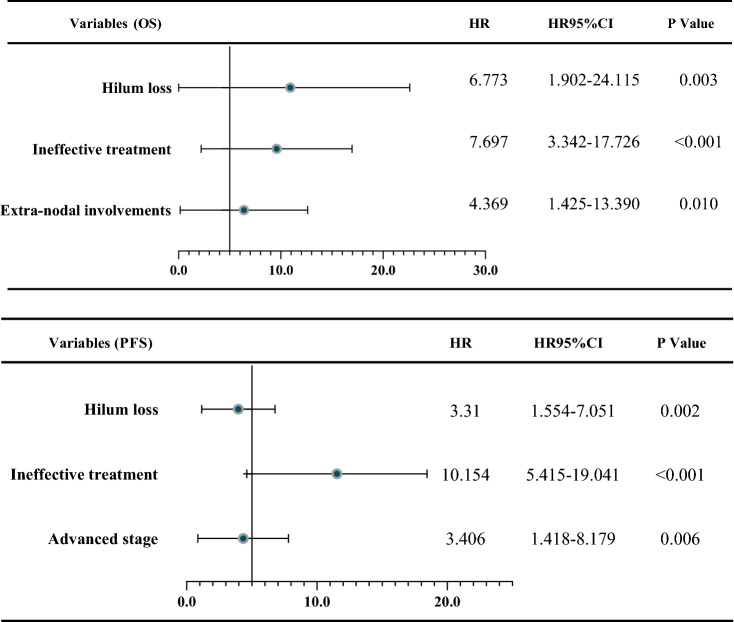
Multivariate Cox regression analyses of PFS and OS.

LDH, lactate dehydrogenase; PFS, progression-free survival; OS, overall survival; HR, hazard ratio; CI, confidence interval.

**Figure 2:**
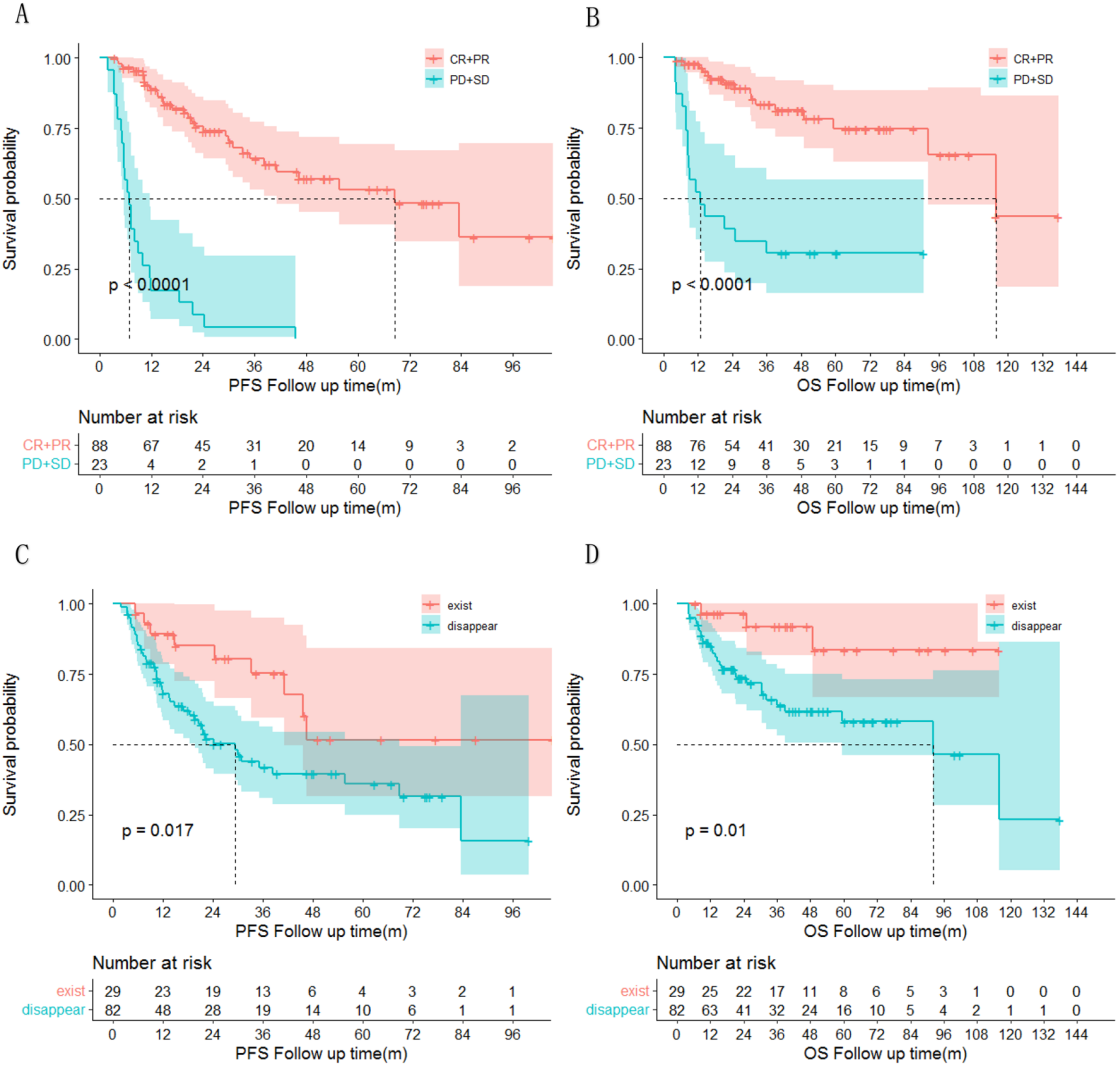
PFS and OS analysis of subgroups with different efficacy and lymphatic hilum performance. CR, complete remission; PD, progressive disease; PR, partial remission; SD, stable disease; PFS, progression-free survival; OS, overall survival.

In the multivariate analysis (Table 3), hilum loss and ineffective treatment were independent risk variables for both PFS and OS (Fig. 2). Specifically, hilum loss (HR, 6.773; 95% CI 1.902 to24.115; *P* = 0.003), extra-nodal involvement (HR, 4.369; 95% CI 1.425 to 13.390; *P* = 0.010) and ineffective treatment (HR, 7.697; 95% CI 3.342 to 17.726; *P* < 0.001) were independently associated with a shorter OS. However, elevated serum LDH level (*P* = 0.064), advanced stage (III–IV) (*P* = 0.374) and decreased HB (*P* = 0.422) were not independent predictors of OS. Among the indicators that affect the PFS, hilum loss (HR, 3.31; 95% CI 1.554 to 7.051; *P* = 0.002), ineffective treatment (HR, 10.154; 95% CI 5.415 to 19.041; *P* < 0.001) and advanced stage (HR, 3.406; 95% CI 1.418 to 8.179; *P* = 0.006) were independent risk factors for shorter PFS.

According to the findings from multivariate analysis, 1-year, 3-year, and 5-year PFS and OS for patients were calculated. 1- and 3-year PFS rate for patients with ineffective treatment was 17.4% and 0%, 1-, 3- and 5-year OS rates were 52.2%, 30.4% and 0%, respectively. In comparison, 3-year and 5-year OS and PFS rate for patients with effective treatment was 83.4%, 74.7% and 64.1%, 53.1%, respectively. For patients with pre-chemotherapy hilum present, PFS and OS at 1-, 3-, and 5- years was 89.3%, 75.2%, 51.5% and 96.4%, 91.8%, 83.5%, respectively. However, for patients with pre-chemotherapy hilum loss, the 1-year, 3-year, and 5-year OS and PFS was 85.0%, 63.8%, 58.1% and 68.0%, 41.8%, 35.9%, respectively.

### Comparison of the new model and the IPI

**Figure 3:**
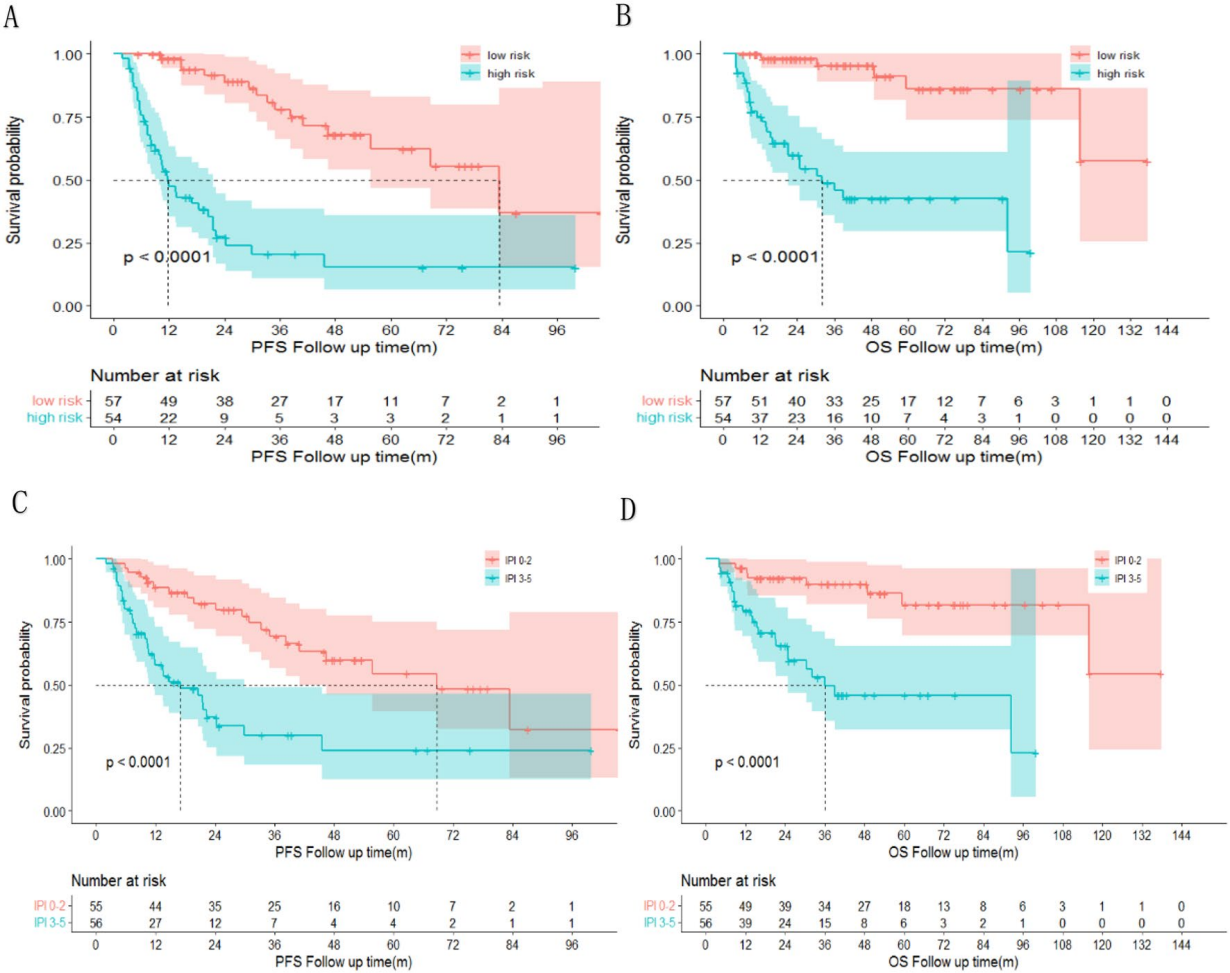
Kaplan–Meier estimated the overall survival of 111 patients with diffuse large b-cell lymphoma based on the risk groups defined by the new model and IPI. PFS, progression-free survival; OS, overall survival. Figures (**A**) and (**B**) show the survival curves for high-risk and low-risk patients in the new model. Figures (**C**) and (**D**) show the survival curves for patients in the better prognosis group (IPI score 0–2) and those in the poorer prognosis group (IPI score 3–5) for the IPI score. It is clear that the new model can better distinguish between different risk groups of people.

A prognostic model for both PFS and OS in DLBCL patients was developed by combining IPI, state after first-line standardized chemotherapy and lymphatic hilum. As recommended in the NCCN guideline, the 5-year OS was 76% for patients with a better prognosis (IPI score 0–2) and 39% for those in the poorer prognosis group (IPI score 3–5) in the IPI score. However, in this study, the 1-, 3- and 5-year OS and PFS in the poorer prognosis group were 79.3 ± 5.6%, 49.5 ± 8.2%, 45.9 ± 8.3% and 57.9 ± 6.9%, 30.0 ± 7.5%, 24.0 ± 8.1%, respectively. The 1-, 3- and 5-year OS and PFS in the better prognosis group were 96.4 ± 2.5%, 89.9 ± 4.3%, 81.8 ± 6.8% and 88.7 ± 4.3%, 69.2 ± 7.0%, 54.4 ± 8.9%, respectively. With the new model, the 1-, 3- and 5-year OS and PFS in the highest risk group was 75.1 ± 6.0%, 48.7 ± 7.6%, 42.6 ± 7.8% and 47.3 ± 7.1%, 20.5 ± 6.6%, 15.3 ± 6.6%. The 1-, 3- and 5-year OS and PFS in the lowest risk group was 98.0 ± 1.9%,95.3 ± 3.3%, 86.1 ± 6.9% and 98.1 ± 1.9%,78.0 ± 6.6%,62.3 ± 9.0%, respectively. (Fig. 3).

**Figure 4:**
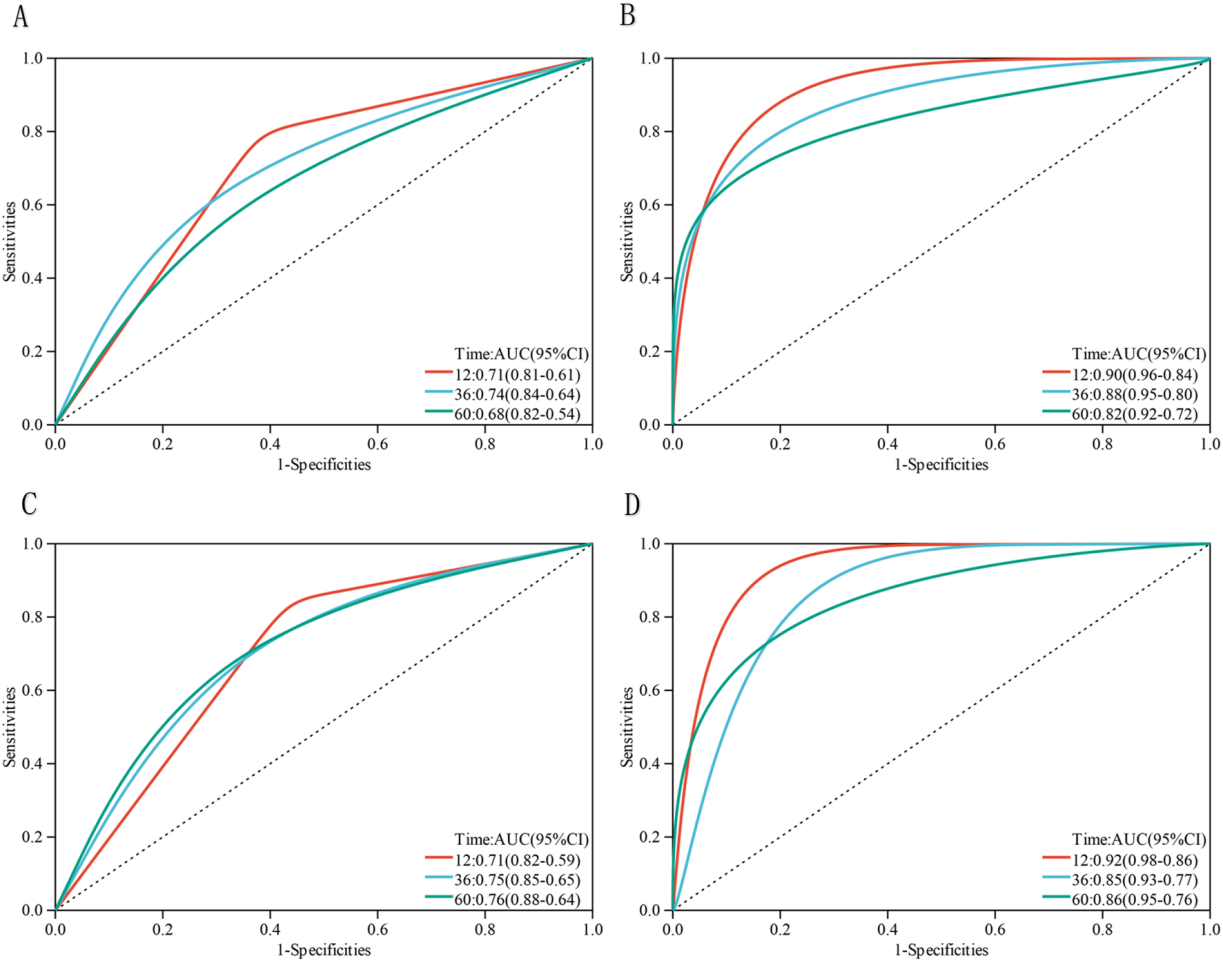
IPI combined with lymphatic hilum disappearance and ineffective treatment is a better prognostic model for PFS (**A**,**B**) and OS (**C**,**D**) in DLBCL. AUC, area under the curve; CI, confidence interval; Time (month).

In this study, the AUCs for IPI alone were 0.71 (95% CI 0.61–0.81), 0.74 (95% CI 0.64–0.84), and 0.68 (95% CI 0.54–0.82) for 1-, 3-, and 5-year PFS, respectively. The AUCs for IPI alone were 0.71 (95% CI 0.59–0.82), 0.75 (95% CI 0.65–0.85) and 0.76 (95% CI 0.64–0.88), for 1-, 3-, and 5-year OS, respectively. And the new model was proved to have better prognostic significance than IPI alone (Fig. 4) [OS: C index, 0.75 (95% CI 0.66–0.84) for the new model vs. 0.69 (95% CI 0.6–0.78) for the IPI alone; PFS: C index, 0.74 (95% CI 0.65–0.83) for the new model vs. 0.67 (95% CI 0.58–0.76) for the IPI alone].

## Discussion

In this study, hilum loss and ineffective treatment remain independent risk factors for shorter PFS and OS with the inclusion of age, stage, entra-nodal involvements and LDH, suggesting that hilum and state after first-line standardized chemotherapy were more powerful predictors than age, stage, extra-nodal involvements and LDH. In this study, a new model about DLBCL was developed by analyzing the effect of ultrasound characteristics on OS and PFS. And the new model was proved to have better prognostic significance than IPI alone (AUC: 0.90, 0.88, and 0.82 vs. 0.71, 0.74, and 0.68 for 1-, 3-, and 5-year PFS, respectively; AUC: 0.92, 0.85 and 0.86 vs. 0.71, 0.75 and 0.76, for 1-, 3-, and 5-year OS, respectively).

Currently, most prediction models are based on basic information about the patient before treatment. However, the response to treatment varies greatly from individual to individual. For some patients, the response to treatment may be the most important determinant of long-term prognosis. The prognosis of patients may be a continuous and dynamic process. Previous studies have confirmed that the rate of chemotherapy remission in lymphoma correlates significantly with patient's indicators related to disease progression^10^ and patients who achieved CR had better outcomes than those who achieved PR or SD^17–20^. Early progression sometimes predicted poor outcomes^21–23^. In this study, ineffective treatment (SD or PD) was the strongest categorical predictor of all the parameters for OS [HR, 6.223; 95% confidence interval (CI), 2.992 to 12.943; *P* < 0.001] and PFS [HR, 10.154; 95% CI 5.415 to 19.041; *P* < 0.001]. Thus, the state after first-line standardized chemotherapy allowed for a good stratification of patients with different prognoses. Similarly, in a study that investigated the prognosis of peripheral T-cell lymphoma, the results indicated that a positive end-of-treatment PET was predictive of both PFS and OS at univariate level, and also for OS at multivariate level adjusted for baseline IPI risk groups^24^. The reasons for this were related to the fact that the ineffective chemotherapy group was mostly advanced, with high IPI scores, high Ki67 expression, and high tumor load indicators before chemotherapy, resulting in residual lesions after the end of chemotherapy. And residual lesions influenced prognosis^25^. Although patients who are SD/PD after standard treatment may achieve CR after other regimens, the long-term prognosis of this group of patients may be very different from that of patients who achieve CR on a single standard treatment. Therefore, the response rates of different individuals to treatment cannot be ignored in the assessment of patient prognostic factors.

**Figure 5:**
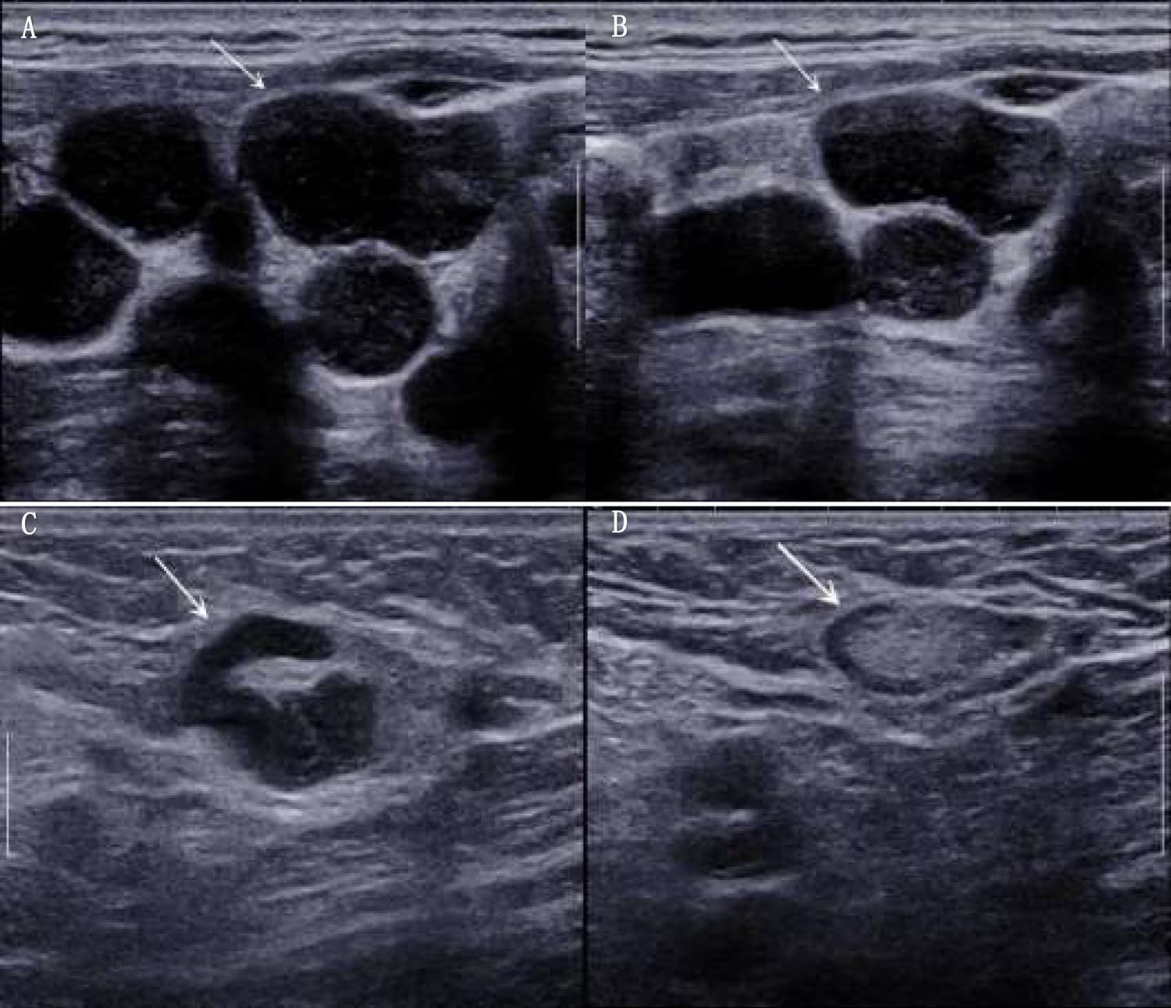
Ultrasound manifestations of the lymphoma. The image on the left is an ultrasound image of the left cervical lymph node in a patient with DLBCL before chemotherapy, showing the loss of lymphatic hilum structures (**A**). On the right side is an image of the lymph node in the same area of the same patient after standardized treatment (**B**). It can be seen that lymphoma with hilum loss is not effective with chemotherapy. However, lymphomas that showed the presence of lymphatic hilum structures on ultrasound decreased significantly in size after standardized chemotherapy, and the morphology of the lymph nodes gradually normalized (**C**,**D**).

Patients who lost the lymphatic hilum before chemotherapy were more likely to have poorer OS (HR, 5.241; 95% CI 1.565 to 17.551; *P* = 0.007) and PFS (HR, 3.31; 95% CI 1.554 to 7.051; *P* = 0.002). The 3-year, and 5-year OS and PFS for patients with lymphatic hilum present before chemotherapy was 91.8%, 83.5% and 75.2%, 51.5%, respectively. And the 3-year, and 5-year OS and PFS for patients with hilum loss was 63.8%, 58.1% and 41.8%, 35.9%, respectively. Obviously, the status of the hilum well distinguished the better prognosis group from the poorer prognosis group (Fig. 5). In previous studies, cortical thickening of lymph nodes and even the disappearance of hilum had been shown to be an important prognostic indicator for the diagnosis of OS in malignancies of the gastrointestinal tract, breast and nasopharynx^26,27^, although the mechanism of lymphatic hilum as a prognostic factor remains unclear. As we know, the hypoechoic lymph node margins are the lymph node cortex in ultrasonography, which is mainly substantial tissue and histologically confirmed to be composed of lymphoid nodule. In contrast, the hyperechoic central of lymph nodes are formed by medullary sinuses, connective tissue, fat and arterial vasculature^28^. Therefore, it is probably because that the blood vessels in the hilum were completely invaded by the tumor tissue and replaced by immature neovascularization. The neovascularization, however, consisted of only two parallel-aligned endothelial cells, which were unable to transport chemotherapeutic drugs, thus reducing the effectiveness of chemotherapy^29^. Consistent with previous findings, our study also suggested that the presence or absence of hilum echogenicity on ultrasonography was highly correlated with patient survival and could be used as a reference indicator to determine prognosis.

On the basis of independent predictors from the multivariate regression, a prognostic model for both PFS and OS in DLBCL patients was developed by combining IPI, state after first-line standardized chemotherapy and lymphatic hilum. OS and PFS were well predicted by the new model. As the results in the survival analysis, the new model in the low-risk range predicted better 1-, 3- and 5-year OS and PFS than those evaluated by the IPI score in the better prognosis range. In the high-risk range, the new model predicted worse 1-, 3- and 5-year OS and PFS than the 5-year OS and PFS evaluated in the poorer prognosis group in the IPI score. Apparently, the new model improved the ability to identify treatment-sensitive patients compared to IPI and also captured more patients at high risk of disease progression and death early. The new model did demonstrate advantages over IPI alone in predicting OS and PFS in DLBCL patients [OS: C index, 0.75 (95% CI 0.66–0.84) for the new model vs. 0.69 (95% CI 0.6–0.78) for the IPI alone; PFS: C index, 0.74 (95% CI 0.65–0.83) for the new model vs. 0.67 (95% CI 0.58–0.76) for the IPI alone]. We therefore consider that for high-risk patients, particularly those with shorter PFS, more aggressive induction therapy, additional radiotherapy, and other new treatment approaches should be considered in future clinical studies. For low-risk patients, standard first-line chemotherapy regimens can produce excellent results.

As a retrospective cohort study, our study had a limited number of subjects and study metrics may have been influenced by many confounding variables beyond our control. Besides, multicenter prospective studies are needed to explore metrics suggesting prognostic information in the future. In addition, our study did not include new techniques such as ultrasound elasticity and ultrasound dynamic contrast enhancement, and many other ultrasound parameters could be included in future studies to predict survival in DLBCL patients. Fourthly, COO subtypes or other genetic classifications may be several important factors affecting the prognosis of DLBCL. Finally, the study of Ultrasonography was only for intra-nodal lymphomas, prognostic analysis with regard to extra-nodal involvement of lymphomas is a direction for future research.

## Methods

### Ethics

As a portion of the study, the First Affiliated Hospital of Nanjing Medical University approved the research under ethics number 2022-SR-058 and written informed consent was obtained from all the patients. All methods were conducted in accordance with the relevant guidelines and regulations. Clinical and laboratory tests were measured in accordance with the principles of the Declaration of Helsinki.

### Study population

111 patients with DLBCL, including 61(55.0%) females and 50 (45.0%) males, were enrolled between January 2010 and January 2021. All patients received a first-line standard R-CHOP or R-CHOP-like regimen with curative intent. The median follow-up time was 115.8 months (range, 4–138 months). Patients were excluded based on the following exclusion criteria: selected lymph nodes without pathological confirmation; Patients with Ann arbor IE staging; patients with previously treated or relapsed lymphoma; patients with other cachexia (deaths due to other causes); incomplete clinical information; and patients who were lost to follow-up. Inclusion criteria: Patients who underwent PET-CT; Lymph nodes with a maximum SUV value; Lymph nodes with core needle biopsy or resection biopsy. Pathological confirmation of diagnosis was performed for all patients based on the WHO 2016 classification of tumors and haematopoietic and lymphoid tissues.

### Clinical data

We retrospectively collected data from the DLBCL patients after first-line standardized chemotherapy. From medical records, age (< 60y, > 60y), gender (male, female), Ann Arbor stage (I-IV), B-symptoms (defined as recurrent fever, night sweats or weight loss > 10%), IPI (Score 0–5), Eastern Cooperative Oncology Group performance status (ECOG PS) (0–4), state after first-line standardized chemotherapy, and treatment regimen were collected. Likewise, laboratorial data were available from the hospital-based laboratory, such as serum β2-microglobulin (serum β2-MG), lactate dehydrogenase (LDH), and hemoglobin (HB). Serum β2-MG was determined by scattering turbidimetric method. According to previous studies, the cutoff values for serum β2-MG, LDH and HB were 2.5 mg/L, 271 U/L and 120 g/L, respectively^30,31^. The IPI score was used to divide all patients into two groups. Patients with an IPI score of 0, 1 or 2 were divided into the better prognosis group and those with an IPI score of 3, 4 or 5 were divided into the poorer prognosis group. State after first-line standardized chemotherapy was based on the 5-Point Deauville Score. Patients were divided into four groups: complete remission (CR), partial remission (PR), stable disease (SD), and progression disease (PD)^32^. Patients with complete and partial remission were classified as the overall response group, while patients with stable disease and progression disease were classified as the ineffective group^33^.

### US data

The Aixplorer ultrasound system (SuperSonic Imagine, Aix-en-Provence, France) equipped with an SL15-4 linear transducer was used for the examination. The patients were placed in a supine position, breathed calmly, and fully exposed the area to be examined. In the ultrasound examination, lymph nodes with a maximum SUV value were recommended for further puncture biopsy. Finally, a total of 111 lymph nodes in 111 patients with definite DLBCL were retrospectively analyzed. Parameters of longitudinal-to-transverse axis ratio (L/T ratio, LT ratio < 2, LT ratio > 2), hilum of lymph nodes (exist, disappear), border (clear, unclear) and trend of fusion of lymphoma (yes, no) were collected. Unlike histologically or anatomically, lymphatic hilum mainly refers to the location of vascular access and lymphatic outflow. On grey-scale ultrasound, lymphatic hilum refers to hyperechoic echogenicity that appears as thin lines, strips, clusters, teardrops or irregular patterns on the sonogram. The lymphatic hilum condition was divided into presence and absence. All the qualitative and quantitative indicators of ultrasonographic imaging were assessed by two radiologists with more than five years of experience.

### Patients’ follow-up

The follow-up data were obtained through electronic medical records and telephone interviews. Statistically, progression-free survival (PFS) is defined as the number of months that pass without progression of the tumor or until death, whichever occurs first. Overall survival (OS) is measured from diagnosis to death or last follow-up^5^.

### Statistical analysis

A descriptive analysis was used to examine the clinicopathologic characteristics of enrolled patients. Kaplan–Meier survival curves and log-rank tests were used to estimate survival time. A median survival time was calculated for each patient, along with its 95% confidence interval (CI). The independent prognostic factors were determined using univariate and multivariate Cox regression analyses. The Cox proportional risk regression model was used to estimate the risk ratio (HR). Calculations of AUC values were made using receiver operating characteristic (ROC) curves. Variables with statistical significance in the multivariate analysis were added to the IPI score to build a new predictive model. The concordance index (C-index) was applied to evaluate the discrimination of the model. A c-statistic of 0.5 to 0.7 was interpreted as low discrimination, 0.7–0.9 as moderate, and > 0.9 as high^34^. Statistical analysis was performed using IBM SPSS Statistical software, Version 25.0 (IBM; Armonk, NY) and the survival ROC package in R, version 4.1.3 (http://www.R-project.org). Unless otherwise stated, *P* < 0.05 was considered statistically significant.

## Data Availability

The datasets generated and analysed during the current study are available from the corresponding author on reasonable request.
